# Fast Triggering of Shape Memory Polymers using an Embedded Carbon Nanotube Sponge Network

**DOI:** 10.1038/srep24148

**Published:** 2016-04-07

**Authors:** Guoxiang Zhou, Heng Zhang, Shuping Xu, Xuchun Gui, Hongqiu Wei, Jinsong Leng, Nikhil Koratkar, Jing Zhong

**Affiliations:** 1 School of Civil Engineering, Wuhan Polytechnic Institute. Wuhan 430023, P. R. China; 2 State Key Lab of Optoelectronic Materials and Technologies, School of Physics and Engineering, Sun Yat-sen University, Guangzhou 510275, P. R. China; 3 Centre for Composite Materials and Structures, Harbin Institute of Technology, Harbin, 150080, P. R. China; 4 Department of Mechanical, Aerospace and Nuclear Engineering, Rensselaer Polytechnic Institute, Troy, New York 12180, USA; 5 School of Civil Engineering, Harbin Institute of Technology. Harbin 150090, P. R. China; 6 Key Lab of Structures Dynamic Behavior and Control, (Harbin Institute of Technology), Ministry of Education, Harbin 150090, Heilongjiang, China

## Abstract

In this work, a 3-D porous carbon nanotube sponge (CNTS) was embedded within a shape memory polymer (SMPs) matrix. We demonstrate complete infiltration and filling of the SMPs into the CNTS by capillary force without any damage to the CNTS structure. With only ~0.2 wt% carbon nanotube loading, the glass transition temperature is increased by ~20 °C, indicating strong interaction between CNTS and the SMPs matrix. Further, we find that the uniform distribution of the carbon nanotubes in the nanocomposite results in high electrical conductivity, and thus highly effective electricity triggering capability. The carbon nanotube sponge shape memory polymer (CNTS/SMPs) nanocomposite could be triggered within ~10 seconds by the application of ~10 volts. Results from finite element simulations showed good agreement with the experimental results, and indicated that for our system the interface thermal energy loss does not have a significant effect on the heating rate of the polymer matrix.

The advancement of smart materials and structures has broad impact on aeronautics, civil engineering construction, gas and steam turbines, as well as wind power generators^1^. Shape memory polymers (SMPs), are one of the most widely used smart materials, mainly due to their adjustable triggering temperature, low cost and facile synthesis^2^. SMPs have already shown great potential in fields as diverse as bio-medical devices, self-healing sealants, and aviation^3^,^4^,^5^.

SMPs can change their shape upon heating. This triggering of the shape change is induced by the lowering of inter-macromolecular movement barriers in the SMPs^6^. Traditionally, SMPs is actuated by direct heating by heat source such as an oven, which limits its practical applications. Therefore new approaches to realize fast and effective remote triggering of SMPs materials are necessary. Adding field sensitive particles, such as magnetic materials and electrically conductive materials, into the SMPs matrix can allow the SMPs composites to be triggered by external field. For example, it has been shown that iron oxide nanoparticle reinforced SMPs nanocomposites can be actuated by magnetic field^7^. The heating in such composite materials is caused by phonon vibrations of the nanoparticles induced by the applied electro-magnetic waves^7^,^8^. However, application of high magnetic fields requires large bulky devices with high power requirement which is not convenient for SMPs triggering.

An alternative approach is to use electrically actuated SMPs composites, which rely on the formation of an inter-connected conductive network of particles in the SMPs. When the content of the conductive particles exceed the percolation threshold, the conductive network can generate joule heating, the efficiency of which depends on the electrical conductivity of the composites. Addition of conductive nano-particles into the SMPs to increase its conductivity has been explored in the literature^9^,^10^,^11^,^12^,^13^. In order to achieve fast triggering of SMPs by electricity, uniform dispersion of the electrically conductive nanoparticles with high loading becomes a prerequisite. For example, Cho and co-workers synthesized electroactive polyurethane SMPs nanocomposite with carbon nanotube (CNT) loading as high as 5 wt%, in which the CNTs were pre-treated by strong acid to improve their compatibility and interfacial bonding with SMPs matrix. The obtained SMPs nanocomposite could be triggered within ~10 seconds when constant applied voltage of ~40 volts. However, the fracture strain was decreased significantly^12^. More recently, Leng and co-workers have systematically investigated various conductive nanomaterials as well as their combinations, to improve the electrical triggering performance of SMPs. They showed that synergistic effects of multi-scale conductive additives can increase the electrical conductivity and thus realize fast actuation. A combination of ~5 wt% carbon nanoparticles and ~2 wt% short carbon fibers resulted in a conductivity as high as 2.32 S/cm, and the SMPs shape change could be induced by application of ~24 volts within ~330 seconds^14^,^15^. In spite of the progress that has been made towards fast actuation of SMPs, the required high loading of conductive materials inevitably deteriorates the deformation capability of the SMPs matrix, which is also a key property for their practical application. Fundamentally, it has been long recognized that the stretchability and electrical conductivity of SMPs nanocomposites are difficult properties to combine. Laminating the SMPs with a conductive heating layer is another approach that has shown some practical potential^14^,^15^. For example, coating of carbon paper on the SMPs surface can allow the electrical triggering of SMPs within ~400 seconds by a voltage of ~16 volts, and further adding carbon nanofibers in the SMPs matrix can decrease the recovery time to ~180 seconds^16^. However, this strategy could also limit the deformation capability of SMPs since the weakly bonded conductive layer are prone to delamination from the SMPs under relatively small tensile/bending strain. Even more, since the heat is diffused from the conductive layer to the SMPs, the heating rates of the SMPs are different for different locations depending on their distance from the interface, which generates internal thermal stress. Different thermal expansion coefficient of the conductive layer coating and the SMPs matrix could also lead to delamination and debonding of the laminated conductive film from the SMPs surface. From the above discussion it is clear that there is a need to develop new approaches to synthesize highly conductive SMPs nanocomposite with low nanoparticles loading. This will ensure that the thermal actuation of the SMPs can be efficiently triggered without compromising on the deformation capability of the SMPs.

In this study, we employed a pre-formed nano-porous carbon nanotubes sponge (CNTS) which is synthesized via chemical vapor deposition (CVD) as a 3-D conductive backbone^17^. The SMPs was uniformly dispersed into the CNTS structure. The uniform dispersion of nanotubes in the SMPs matrix results in large changes to the glass transition temperature of the SMPs even at carbon nanotube loadings as low as ~0.2 wt%. The obtained SMPs/CNTS nanocomposites showed exceptionally fast triggering response compared with what has been reported in the literature could be triggered within 10 seconds by a voltage of 10 volts, and less than 2 seconds by 20 volts^12^,^13^,^14^,^15^,^16^. Finite element simulations were also carried out and showed good agreement with the experimental results. The simulation results also indicated that the interface thermal diffusion layer has marginal effect on the heat transport from the CNTS backbone to the SMPs matrix.

## Results and Discussion

### Characterization of the SMPs-CNTS Composite

**Figure 1:**
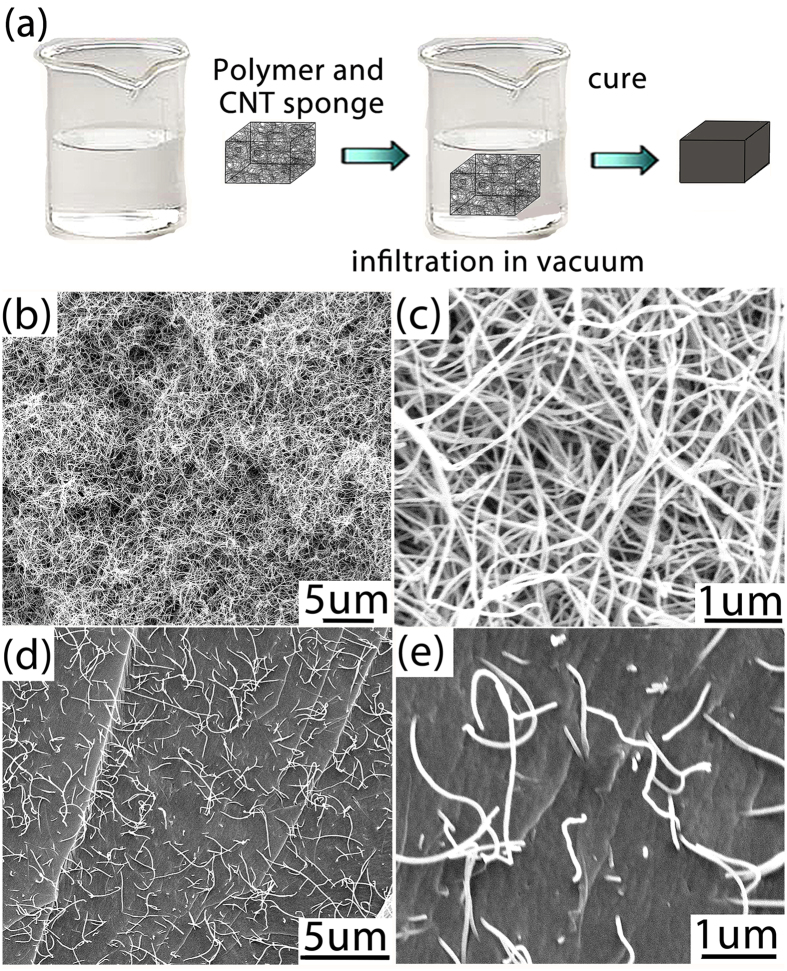
Fabrication of CNT sponge polymer composites (**a**) SEM images of CNTS (**b,c**) and CNTS/SMPs nanocomposite (**d,e**).

The infiltration of the SMPs into the CNTS structure is shown in Fig. 1(a). Figure 1(b,c) shows SEM images of the cross-section of CNTS/SMPs nanocomposites, showing uniform infiltration of the SMPs into the pores of the CNTS. The pores in the CNTS are formed by randomly distributed carbon nanotubes with length of several microns. The average pore size of CNTS used in this study is ~80 nm. Such length scale for the pores can induce significant capillary force without blocking the liquid SMPs precursor, and allowing the precursor to infiltrate into the CNTS. Due to the relatively strong interaction between individual nanotubes in the sponge, the capillary forces associated with polymer filling do not induce any visible collapse of the CNTS, which is frequently observed in the case of CNT forests^17^. In this way, the outstanding electrical conductivity of the CNTS can be successfully inherited by the CNTS/SMPs nanocomposite^18^. Such a nanocomposite structure is in sharp contrast with CNT paper or CNT forest, both of which have been employed as conductive additives to reinforce polymer or SMPs. CNT paper is constituted with randomly piled nanotubes with typical porosities less than 60%^19^. Further, the individual nanotubes are loosely connected in conventional CNT paper, which compromises the mechanical integrity of the structure. The CNT forest, on the other hand, consists of an aligned array of carbon nanotubes. It is well established that weak interaction between individual nanotubes in a CNT forest cannot resist the capillary force induced by the infiltration of liquid, leading to non-uniform distribution of CNTs in the polymer matrix^17^. By contrast, the free-standing CNTS employed in this study is structurally robust, although its porosity (>99%) is much higher than both CNT paper and CNT forest. Figure 1(d,e) shows that the carbon nanotubes in the CNTS are dispersed uniformly in the SMPs matrix without any visible pores, and the nanotubes that protrude out of the matrix can still maintain their inter-connection, which indicates the structural integrity of the CNTS network structure^20^,^21^,^22^,^23^. This strategy is different from that of directly mixing carbon nanotubes into the SMPs matrix, in which low electrical conductivity is achieved even at high nanotube loading (less than ~10^−2 ^S/cm at several wt% of carbon nanotubes)^9^,^10^,^11^,^12^,^13^. The CNT loading in our CNTS/SMPs nanocomposite is as low as ~0.2 wt%, however, its electrical conductivity was measured to be ~1.29 S/cm which is two orders of magnitude higher than the conventional approach of randomly mixing carbon nanotubes in the SMPs matrix.

### Thermal properties of the composites

**Figure 2:**
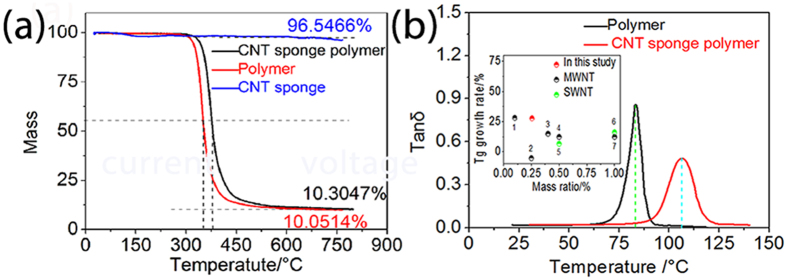
(**a**) TG test of CNT sponge and CNT sponge polymer and (**b**) Glassy transition temperature of pure SMPs and CNTS/SMPs nanocomposite.

Figure 2a presents the effects of the CNTS on the thermal stability of the SMPs. The CNTS significantly improves the stability of the polymer, with the degradation temperature increasing by ~20 °C. The glass transition temperature (Tg), which is an indicator for the triggering temperature, increased from ~83 °C for the pure SMPs to ~106 °C for the CNTS/SMPs nanocomposite (Fig. 2b). The improvement in thermal stability of the SMPs induced by the CNT sponge is one of the highest reported to date (Inset of Fig. 2b)^24^,^25^,^26^,^27^,^28^,^29^,^30^. This is presumably a result of the uniform dispersion of carbon nanotubes in the SMPs matrix, as well as the strong interaction between the nanotubes in the sponge and the SMPs matrix. The presence of CNT can modify the structure, chain mobility and conformation of the polymer chains in the interphase region which typically varies from 10–100 nm from the CNT surface. This overlaps well with the average pore size of ~80 nm in the CNTS which explains the large increase in glass transition temperature that we report.

### Electrical actuation shape memory effects of the composites

The measured electrical conductivity of the CNTS (without polymer infiltration) and the CNTS/SMPs composite are ~1.32 S/cm and ~1.29 S/cm, respectively. Such nearly identical conductivity of the CNTS before and after SMPs polymer infiltration confirms that the 3-D structure as well as the inter-connections between the nanotubes in the CNTS network are well preserved even after the polymer infiltration. To the best of our knowledge, the electrical conductivity of our CNTS/SMPs composite material is the highest among all of the electrically actuated SMPss reported to date in the literature.

**Figure 3:**
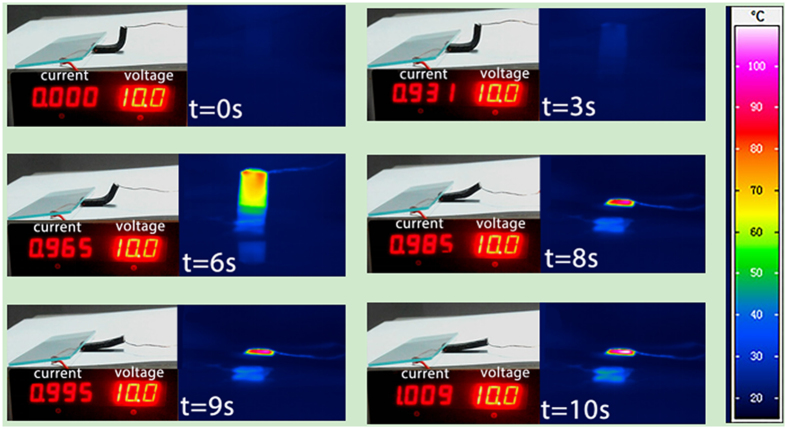
Electrical actuation of CNTS/SMPs nanocomposite with the temperature distribution monitored.

**Figure 4:**
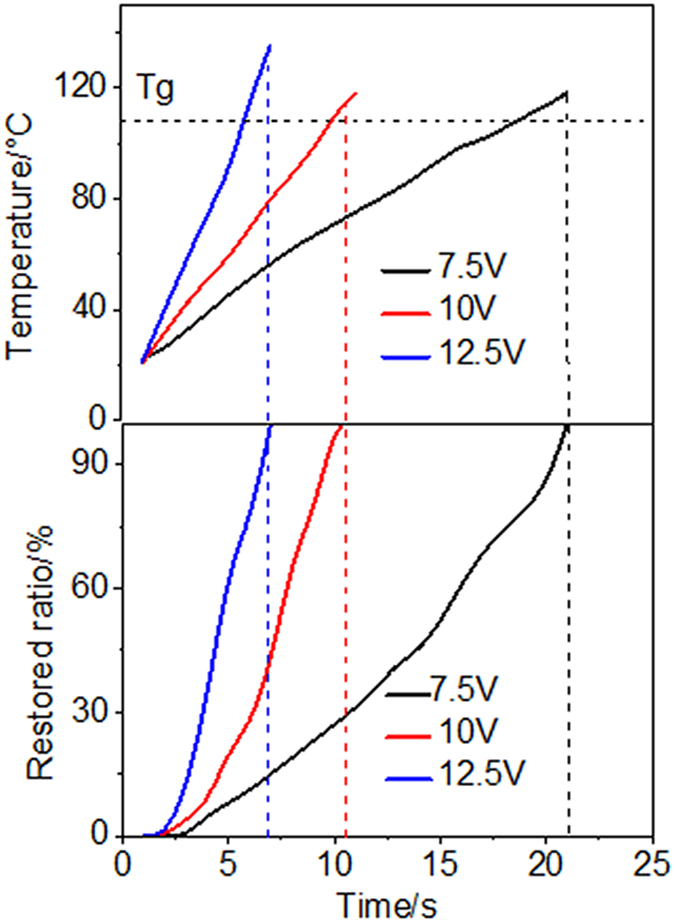
Shape recoverability of CNT sponge polymer under different voltages.

Figure 3 shows the shape memory recovery process and temperature distribution during the electrical actuation of the nanocomposite. The quantitative behavior of the shape recovery process is provided in Fig. 4. The infrared thermal images in Fig. 3 illustrate that the temperature distribution is not uniform with the region near to the silver paste exhibiting the fastest temperature rise. This is because the silver paste itself has very low electrical resistance, compared to the bulk nanocomposite. Triggered by a voltage of 7.5 V (Fig. 4), it takes ~21 seconds (recovery time) for the CNTS/SMPs nanocomposite to completely recover to its original shape. Increasing the triggering voltage to ~10 V decreases the recovery time to ~10 seconds, which is one of the fastest electricity actuated SMPs reported to date. Interestingly, it is noticed that for the cases of 7.5 V and 10 V triggering voltage, when the samples recover to their original shape, the temperature for both cases is ~130 °C, which corresponds to the starting point of the rubbery state of the polymer matrix. However, we find that with further increase of the triggering voltage to ~12.5 V, the temperature corresponding to the point when the sample recovers completely is now significantly greater than 130 °C. Therefore more in-depth study into how the CNTS network affects the relaxation time and viscoelastic properties of the nanocomposite material is warranted and should be the subject of future work.

### FEM model

**Figure 5:**
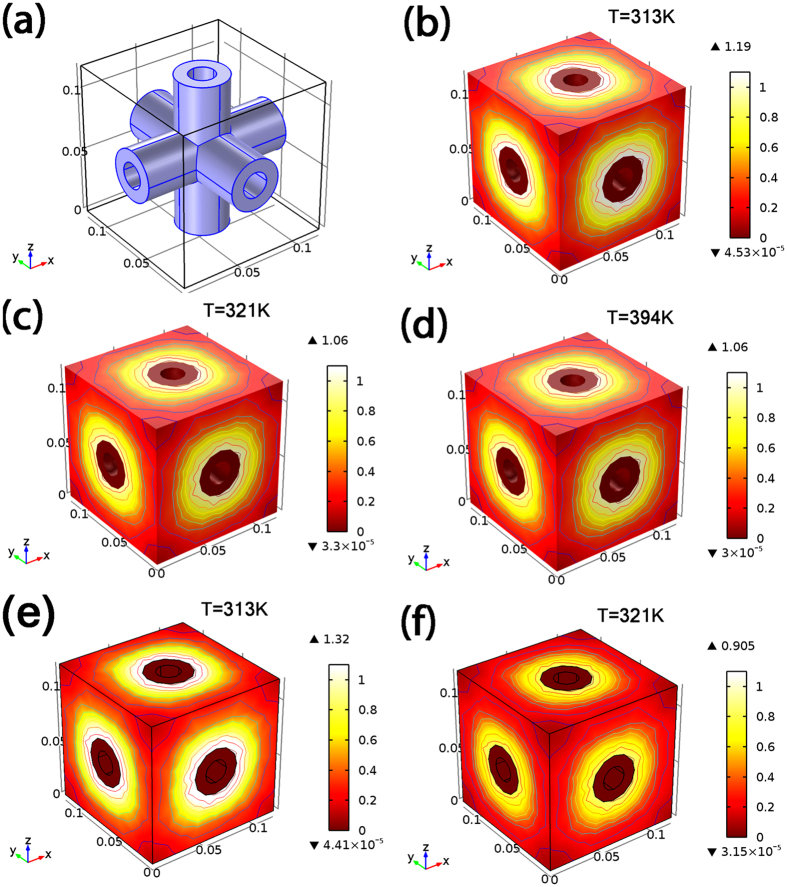
Finite element simulation. Geometric model of typical element (**a**) Distribution of temperature gradient at time 0 s (**b**), 1 s (c), 10 s (**d**) with interface thermal diffusion conductance of 8.3 × 10^−5^. Distribution of temperature gradient at time 0 s (**e**), 1 s (**f**) with interface thermal diffusion conductance of 8.3 × 10^−8^.

In order to understand the joule heating and thermal transport process during the electrical triggering, Finite Elment Method (FEM) simulations were carried out. The model shown in Fig. 5(a) was used to represent the 3-D CNTS structure. On passing electrical current through the CNTS, joule heating enables the CNTS to act as a heating source to diffuse its thermal energy to the surrounding polymer matrix. It can be seen from Fig. 5(b–d), that it takes ~10 s for the nanocomposite to reach a temperature above its Tg, which shows good agreement with the experimental results. The temperature gradient remains relatively stable during the heating process with the interface region displaying the highest gradient. In order to study the effects of the interface layer on the heat diffusion, we decreased the thermal diffusion conductance of the interface layer by three orders (Fig. 5e,f), which, however, did not induce any noticeable difference of heating and temperature gradient. This result is in contrast with the case of thermal transport in nanocomposite materials where an external heat resource is applied. In that case, the nanofiller-polymer interface layer is the primary cause of phonon scattering, which limits the thermal conductivity enhancement.This difference is possibly because in our study, the CNTS acts as a direct and local heat source which reduces the role of the interfacial thermal resistance. The small pore size (~80 nm) in the CNTS also limits the volume of the polymer to be heated and enables rapid and uniform heating to be accomplished.

## Conclusions

Taking advantage of the unique structure/properties of 3D porous CNTS, we synthesized 3D CNTS/SMPs nanocomposites with very high electrical conductivity at low CNT loading, and realized their fast electrical actuation. The CNTS/SMPs nanocomposite could be triggered within ~10 seconds by the application of ~10 volts. The thermal stability and glass transition temperature were both improved, due to the uniform dispersion of the nanotubes and strong interaction between the nanotubes and the SMPs matrix. We employed finite element modeling to simulate electrical generated joule heating, the heat transport process and the temperature distribution. The simulation results agree well with the experiments and also reveal that the interface thermal conductance (at the SMPs-CNTS interface) has a marginal effect on the heating process of the SMPs matrix.

## Methods

### Materials

**Figure 6:**
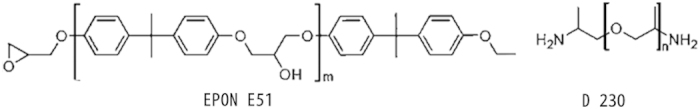
Molecular Formula of E51 and D230.

The CNTS were synthesized by CVD process using ferrocene and 1,2-dichlorobenzene as the catalyst precursor and carbon source, respectively^31^. Ferrocene powders were dissolved in dichlorobenzene to make a solution at a concentration of ~60 mg/ml, which was then continuously injected into a 2-inch quartz tube housed in a resistive furnace by a syringe pump at a feeding rate of ~0.13 ml/min. The reaction temperature was set as ~860 °C. The carrier gas (a mixture of Ar and H_2_) was flowing at a rate of ~2000 ml/min and ~300 ml/min, respectively. A 2 inch × 1 inch quartz sheet was placed in the reaction zone as the growth substrate. The sponge-like products were collected from the quartz substrate after CVD, which typically reach a thickness of 0.8 to 1 cm for a growth period of ~4 hours. The bulk density of Carbon nanotubes sponge is about 5–10 mg/cm^3^ with a specific surface area of 300 to 400 m^2^/g and an average pore size of about 80 nm (porosity of >99%). Epoxy resin E51 (WSR618) was purchased from Nantong Xingchen Synthetic Material Co Ltd. The hardener D230 (C_3n+3_H_6n+10_O_n_N_2_) was purchased from Huntsman. All the materials were used as received without further treatment. The Molecular Formula of E51 (m=0.14) and D230 (n~2.5) are shown in Fig. 6.

### Preparation of CNTS/SMPs nanocomposite

About 10 grams of Epoxy resin was mixed with ~3.2 grams of D230 in vacuum oven with stirring at ~50 °C to lower its viscosity without accelerating the curing process significantly. The pre-formed CNTS was immersed into the mixed liquid, which is then transferred to a vacuum oven at ~50 °C for ~1 hour to facilitate the polymer infiltration process. Then the nanocomposite was cured at ~75 °C for ~4 hours in a vacuum environment.

### Characterization of materials

A scanning electron microscope (SEM) was used to check the microstructures and CNT dispersion quality in the SMPs matrix. The glass transition temperature was investigated by a dynamic mechanical analysis (DMA, TA Q800), with temperature scanning from ~25 to ~140 °C. The heating rate and frequency were set at ~5 °C/min and ~5 Hz, respectively. The dimension of the samples were ~38 *mm* × 5 *mm* × 2 *mm*. The thermal stability was tested by thermogravimetric analysis (TGA) from ~50 to ~800 °C with a heating rate of ~10 °C/min in Ar. The SMPs electrical triggering behaviors were tested using a sample with a dimension of ~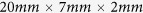. The sample was first heated to ~120 °C at a heating rate of ~10 °C/min from room temperature, and kept at ~120 °C for ~10 mins to ensure uniform temperature distribution. Then the sample was bent around a small cylinder with a diameter of ~20 mm, by an angle of ~90°, followed by cooling to room temperature. The obtained programmed sample was connected to a power source using silver paint to establish electrical contact with the sample surface. Both the temperature distribution and recovery process were monitored by an infrared thermal camera. The restored ratio was defined as: 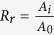, where 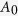 is the initial curvature angle of the sample and 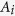 is the angle of the bent sample at time i.

### Finite element model (FEM)

A three dimensional (3D) finite electric thermos model was built using COMSOL Multiphysics 5.1 to analyze the heat transfer process induced by Joule heating of the CNTS^32^,^33^. The CNTS was idealized as a 3D truss structure in this model. The dimension of the 3D model is ~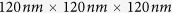. The parameters used in the FEM model are listed in Table 1. In the model an electric field was applied to the CNTS to generate a corresponding temperature field associated with Joule heating. To model thermal losses across the CNTS/polymer interface, a thin film with adjustable low thermal diffusion conductance was inserted between the CNTS and the SMPs matrix and transient response in the 0 to 10 seconds range was investigated.

## Additional Information

**How to cite this article**: Zhou, G. *et al*. Fast Triggering of Shape Memory Polymers using an Embedded Carbon Nanotube Sponge Network. *Sci. Rep.* **6**, 24148; doi: 10.1038/srep24148 (2016).

## Figures and Tables

**Table 1:** Parameters used in FEM modeling of heat transfer.

Parameters	Value	Unit
Voltage	6 × 10^−5^	V
Conductivity (CNT)	1.29	S/cm
Specific heat capacity (CNT)	0.85	J/(g·k)
Specific heat capacity (Polymer)	0.50	J/(g·k)
Inner diameter (CNT)	20	nm
Outer diameter (CNT)	40	nm
Separation distance of CNTs	120	nm
Film layer thermal resistance	8.3 × 10^−5^	*K* · *m*^2^/*W*
Thermal conductivity (CNT)	1000	W/(m·k)
Thermal conductivity (Polymer)	0.2	W/(m·k)
